# The Montpellier Oath

**Published:** 1874-12

**Authors:** 


The Montpellier Oath.—The following oath was taken by young physicians at Montpellier. It was at Montpellier that Rabelais was professor, as also Rondelet, the father of modern zoology:

"I, ..... ......, before the statue of Hippocrates, and in presence of the professors of this school, and of my dear fellow-students, do, in the name of the Most High, swear to be faithful to the laws of honor and probity, in the practice of medicine.

"I will attend the poor gratuitously, and never will I exact more pay than my work is worth. When called to visit families, my eyes shall not see what there takes place; my tongue shall keep silent on the secrets confided to me, and my profession shall never serve for the corruption of society, or in the furthering of crime.

"If I am faithful to my oath, may men honor me; may I be covered with disgrace and scorned by my associates, if I fail." *Nashville Jour. Med. & Surg.*

